# Hydrodynamic Mechanisms Underlying the Burying Behavior of Benthic Fishes: Numerical Simulation and Orthogonal Experimental Study

**DOI:** 10.3390/biomimetics11010055

**Published:** 2026-01-08

**Authors:** Hualong Xie, Xiangxiang Wang, Min Li, Yubin Wang, Fei Xing

**Affiliations:** 1 School of Mechanical Engineering and Automation, Northeastern University, Shenyang 110169, China; 2 Mining Hydraulic Technology and Equipment Engineering Research Center, Liaoning Technical University, Fuxin 123000, China

**Keywords:** self-burial behavior, benthic fish, fluid-particle interaction, sediment fluidization, orthogonal experiment

## Abstract

To avoid predators, benthic fish will stir up the sediment on the seabed by flapping their pectoral fins, thus burying themselves. This self-burial concealment strategy can offer bionic enlightenment for the benthic residence method of Unmanned Underwater Vehicles (UUVs). In this paper, based on the observation results of the self-burial behavior of benthic fish, a two-dimensional fluid-particle numerical model was developed to simulate the processes of sediment transport induced by pectoral fin flapping. In addition, an orthogonal experimental design was employed to analyze the effects of body length, flapping amplitude, flapping number, flapping frequency, and particle size on burial ratio, input power, and burial efficiency. The results reveal that rapid pectoral fin flapping enables benthic fish to fluidize sediments and achieve self-burial. Among the influencing factors, body size has the most significant impact on coverage ratio and input power, as larger fish generate stronger tip vortices and fluid disturbances, making local flow velocities more likely to exceed the critical starting velocity. In contrast, particle size has the weakest effect on burial performance, while kinematic parameters exert a far greater impact on self-burial than environmental parameters. The research results can offer references for the biomimetic design of self-burying UUVs.

## 1. Introduction

Benthic fish refer to fish species that inhabit the bottom layers of marine or freshwater environments, whose physiological structures and behaviors are highly adapted to benthic living. Common species include *Pleuronectiformes*, *Rays*, *Pleuronectes platessa*, *Anguilla*, *Anglerfish*, and *Soles* [1,2]. Typical characteristics of these fish include flattened or streamlined bodies, often with protective colours or markings resembling the surrounding sediments, and in some cases, the evolution of specialized organs such as bioluminescent lures (e.g., *Anglerfish*) or barbels (e.g., *eel*) for environmental sensing or prey attraction. Notably, most benthic fish have evolved a unique burrowing behavior [3], whereby they bury part or all of their bodies into sediment substrates by body undulations, jetting, or fin stirring, forming a hidden state integrated with the surrounding environment.

The primary purposes of burrowing behavior are predation and survival. From a defensive perspective, benthic fish can effectively evade predators’ visual detection by burrowing. For instance, *Pleuronectiformes* rapidly flap their pectoral fins to stir up sediments and settle into soft substrates when sensing danger, leaving only protruding eyes exposed for environmental monitoring [4], while *sand lances* burrow vertically into the sand [5]. From an offensive standpoint, burrowing behavior is often combined with ambush predation strategies. For example, *Scophthalmus maximus* rapidly flaps its pectoral fins to fluidize sediments, after instantaneous burial, waits for the prey to approach, and then leaps out at lightning speed to carry out the hunt [6]. *Anglerfish* use their bioluminescent lure to attract curious small fish, complete precision hunting [7,8]. Many other benthic fish exhibit similar burrowing behaviors, such as *Solea* [9,10], *Pleuronectes platessa* [11,12], *Trypauchen vagina* [13], *Anguilla anguilla* [14], and *Wrasses* [15].

The Unmanned Underwater Vehicle (UUV) is an intelligent autonomous equipment that can complete underwater tasks autonomously, widely used in marine science research, resource exploration, military reconnaissance, and environmental monitoring [16]. UUVs rely on their own energy and control systems to perform tasks, which imposes high requirements on their ability to maintain dynamic balance and positional stability during hovering fixed-point detection [17,18]. Whether it is a hovering UUV through buoyancy adjustment or a hovering UUV relying on auxiliary thrusters, it is necessary to overcome an unfavorable factor in the ocean bottom environment, ocean current. The submarine current velocity is usually between 0.5–1.5 m/s. The ocean current has strong time-varying and spatial heterogeneity. UUV is extremely sensitive to this unpredictable ocean current disturbance, resulting in unexpected drift and affecting the stable detection ability of UUV [19,20]. Moreover, the dynamics models of UUV exhibit highly nonlinear and strongly coupled characteristics, involving the combined effects of fluid inertia, viscous drag, and added mass effects [21,22,23,24]. In addition, time-varying underwater disturbances (e.g., turbulent vortices [25] and density stratification [26]) and uncertain hydrodynamic parameters [27,28] (e.g., varying Reynolds numbers) make it difficult to achieve robust control of UUVs [29,30]. Consequently, environmental unpredictability, coupled with nonlinear vehicle dynamics and parameter uncertainties of UUVs, limits the reliability and efficiency of UUVs in carrying out tasks in benthic environments. Achieving stable benthic station-keeping thus remains a formidable task.

To address the issue of UUV benthic stable residence, some researchers have proposed several strategies. Wang et al. [31] and Matsuda et al. [32] have developed a variable buoyancy system based on ballast water, which enables UUVs to settle on the seabed to extend endurance. Zhang et al. [33] designed a landing gear that enables rigid contact with the seabed, but this approach requires precise terrain matching and often fails in rough or soft substrates. Furthermore, the mounting bracket significantly increases the volume and weight of the UUV, reducing the flexibility to perform tasks in other environments. In addition, many researchers [19,22,34] employed anchor-chain systems to enhance hovering stability. While the anchor chain system can enhance the anti-current capability, in order to avoid seabed collisions between the UUV and the seabed, UUVs equipped with the anchor chain systems require frequent thruster activation to correct their positions. Therefore, the improvement of hovering stability by the anchor chain system comes at the expense of maneuverability, endurance, and payload capacity. More critically, these methods substantially reduce the stealth of UUV, so that it can be easily found by acoustic detection equipment when it resides on the seabed. Thus, existing solutions fail to achieve an optimal balance between stability, adaptability, and concealment. There is still room for improvement in benthic residence technology, and more flexible and efficient approaches are needed to meet future mission requirements.

The burying behavior of benthic fishes serves as a biomimetic reference for enabling stable benthic dwelling of UUVs. Inspired by such strategies, UUVs can realize self-burial and stable seabed settlement. This strategy confers twofold benefits:

First, enhancement of concealment. Acoustic detection of buried targets on the seabed is still a global challenge [35,36], as the acoustic attenuation coefficient in sandy medium is 2–4 orders of magnitude higher than in seawater. For example, the absorption attenuation of a 100 kHz acoustic signal is about 0.031 dB/m in seawater [37], but reaches 74 dB/m in sandy sediments [38]. The strong attenuation characteristics of sound waves in seabed sediments make it extremely difficult to detect and identify buried targets on the seabed. Therefore, the buried UUV can effectively avoid the detection of acoustic equipment and significantly enhance its underwater covert operation capabilities.

Second, extension of endurance. The UUV in its buried state can stably dwell on the seabed for reconnaissance without the need to expend energy counteracting ocean currents, thereby markedly prolonging its operational endurance.

Currently, most studies on fish burrowing behavior remain at the level of biological observation. Lu et al. [39] observed the preference of *Paralichthys olivaceus* juveniles for benthic particle size, and found that juveniles preferred to bury in fine sand, and the success rate of burial in fine sand was as high as 93.1%. Corn et al. [40] found that *Parophrys vetulus* uses the periodic fluctuation of its body to force water flow to disturb the bottom sediment and fluidize the sediment particles. The fluid vortex carries the sediment particles to reach the top of the fish’s body. When the fish stops fluctuating, the fluid velocity above the fish decreases, and the sediment particles settle onto the fish’s surface, achieving the burial behavior. They also quantified the effects of sediment grain size and fish body size on burial behavior, concluding that sand particle size had little impact, while larger fish typically buried using lower frequencies and longer durations. Gibson et al. [12] studied the sediment selection behavior of juvenile *Pleuronectes platessa* of different individual sizes, and found that smaller fish prefer finer-grained sediments, while larger fish showed no significant preference for the particle size of sediments. Scott et al. [41,42] employed Particle Image Velocimetry (PIV) and video analysis to investigate *Potamotrygon motoro* burial behavior on sediments. *Potamotrygon motoro* fluidizes the sediment below the fish’s body and transports it to the top of the pectoral fin by repeatedly and rapidly swinging the pectoral fin. The water–sand vortex collides above the midline of the stingray’s back, and the sediment settles to the back of the fish’s body, thus completing the burial behavior. Subsequently, they discussed the influence of the flapping frequency and amplitude of the fish body on the burial behavior. With the increase in the flapping displacement and velocity of the pectoral fin, the sediment coverage area on the back of the fish’s body also increased accordingly. The average sediment coverage area is about 82.5% of the back area of the fish body.

Biological observation experiments can analyze the burial process of fish on a macro scale. However, the mechanical parameters of live fish are difficult to measure accurately, and their burying behavior cannot be artificially controlled. Therefore, some researchers have employed controllable simulation models to conduct burying studies. To reproduce the burial behavior of benthic fish through experiments, McKee et al. [43] constructed a variable-speed-driven elliptical silica gel plate model to study the impact of the fluctuation frequency of the *Pleuronectiformes* model on the burrowing behavior. The study shows that the coverage area of the sediment increases with the increase in the model’s oscillation frequency. The flapping frequency required to completely cover the model exceeds 10 Hz, but the flapping frequency required for the complete burial of a live *Pleuronectiformes* is usually within 10 Hz.

The present study aims to reveal the hydrodynamic mechanisms of benthic fish burrowing through numerical simulations and provide insights for biomimetic self-buried UUV design. First, a two-dimensional fluid-particle coupling model of self-burial behavior was developed to reproduce sediment fluidization, transport, and burial under fin flapping based on biological observation results of fish flapping burial behavior. Second, an orthogonal design was used to examine the effects of geometric parameters (body length), kinematic parameters (flapping amplitude, number, and frequency), and environmental parameters (particle size) on self-burial coverage ratio, input power, and burial efficiency. Combined with the velocity distribution, pressure field distribution, and vorticity structure evolution of the flow field, the synergistic mechanism of wingtip vortex, pressure difference, and fluid disturbance in particle initiation, transport, and deposition during the burial process was revealed. Finally, the enlightenment of benthic fish self-burial behavior on the design of underwater bionic self-burying UUVs is discussed, including the matching strategy of wing span size and motion parameters, energy consumption optimization schemes, and adaptability to different sediment environments.

The paper is organized as follows: Section 2 presents the problem formulation and hydrodynamic evaluation parameters. Section 3 first introduces the specific implementation details of the numerical model and then uses a standard example to verify the accuracy of the method, and verifies the grid and time step independence of the numerical model in the paper. Section 4 gives the specific scheme of the orthogonal experiment. Section 5 discusses the results of the orthogonal test and analyzes the numerical simulation results of this paper. Section 6 discusses the correlation between the numerical results of this article and the biological observation results. Section 7 presents the conclusions of the study.

## 2. Problem Formulation and Hydrodynamic Parameters

### 2.1. Problem Formulation

**Figure 1:**
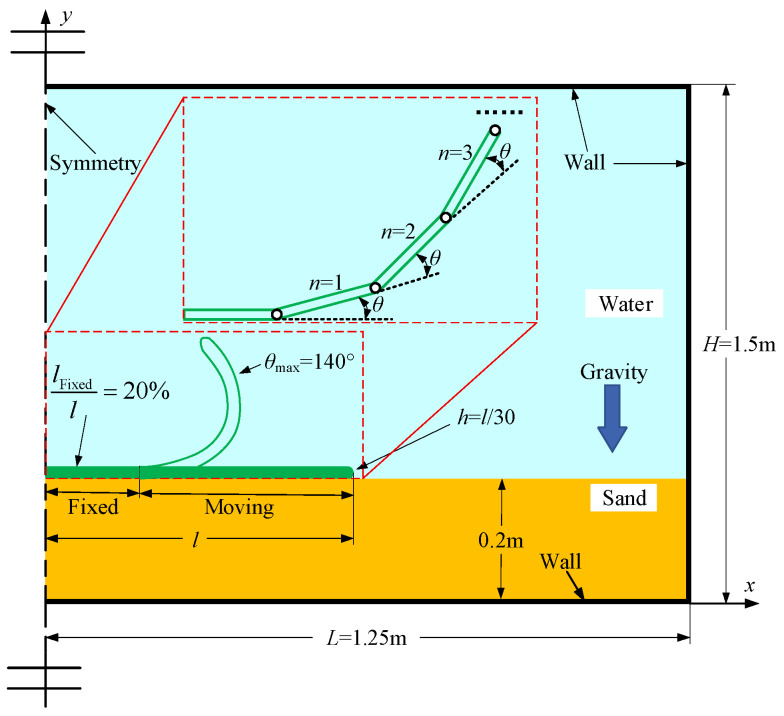
Schematic diagram of the numerical computational model of the burying behavior of benthic fish.

Figure 1 illustrates the two-dimensional numerical calculation model of the self-burial behavior of benthic fish. The computational domain has a length *L* = 1.25 m and a height *H* = 1.5 m. The bottom of the domain is filled with particles, while the remaining region is occupied by fluid. At the initial moment, the particle layer thickness is 0.2 m, with a particle density *ρ*_p_ = 2500 kg/m^3^. The particle diameters *d*_p_ are set to 0.1 mm, 0.3 mm, 0.5 mm, 0.7 mm, and 0.9 mm, respectively. The initial particle volume fraction *θ*_p-initial_ is 63%, and the particle dynamic viscosity is *v*_p_ = 20 kg/(m·s). The fluid has a density of *ρ*_f_ = 998.2 kg/m^3^ and fluid dynamic viscosity of *v*_f_ = 1.003 × 10^−3^ kg/(m·s). Since the cross-sectional profile of benthic fish is typically irregular. In order to simplify the calculation model, the cross-section of the fish body is simplified to a plate with a length of *l* and a thickness *h* = *l*/30. The plate lengths *l* are set to 3 cm, 6 cm, 9 cm, 12 cm, and 15 cm, respectively, covering the size range from juvenile to adult fish. Initially, the plate is positioned above the particle layer. Based on the fish’s locomotion, the plate is divided into a fixed portion and a moving portion: the fixed part represents the rigid skeletal structure of the body, while the moving part corresponds to the flexible cartilaginous pectoral fins. The fixed portion accounts for 20% of the plate length.

According to the observations of Seamone et al. [41,42] on ray self-burial, when rays are buried, the left and right pectoral fins move synchronously, exhibiting pronounced symmetry. Therefore, the left boundary of the computational domain is specified as a symmetry boundary, while the other boundaries are set as walls.

The moving portion of the plate is divided into *n* consecutive equal-length joint units, with each joint unit forming an angle *θ* relative to its preceding joint unit. The joint unit closer to the body exhibits the smallest amplitude of motion, while the distal joint unit exhibits the largest amplitude. To generate a nearly smooth bending deformation of the plate, the number of joint units is set to *n* = 20. Consequently, the distal joint unit forms an angle of *n*·*θ*° with the *x*-axis, and the maximum bending angle of the plate *θ*_max_ = *n*·*θ*°.

For a complete motion burial process, the plate starts to move from its initial position, bends to the maximum angle, and then returns to the initial position, which is defined as one flapping cycle. The number of flaps is denoted as *N*. According to the observations of fish burial by Seamone et al. [41,42], fish typically require 5–6 flapping cycles to complete the burial process, with a flapping frequency of approximately 3–5 Hz. Therefore, in this study, the flapping cycle numbers *N* are set to 2, 4, 6, 8, and 10, and the flapping frequencies *f* are set to 5 Hz, 6 Hz, 7 Hz, 8 Hz, and 9 Hz, respectively. The summarized parameters of the numerical model are listed in Table 1.

### 2.2. Hydrodynamic Parameters

To evaluate the self-burial effect of each factor in the present numerical model, the coverage ratio, input power, and burial efficiency are defined as evaluation indices. These metrics are used to evaluate the impact of plate length, flapping frequency, flapping cycles, flapping amplitude, and particle size on the self-burial effect and its primary and secondary orders.

The coverage ratio is defined as the ratio of the area of the plate surface covered by particles at the end of the burial process to the total surface area of the plate. A coverage ratio of 1 indicates that particles completely cover the plate surface, whereas a value of 0 indicates no particle coverage. The coverage ratio thus reflects the percentage of the fish body surface that is covered by sediment upon completion of the burial action. The coverage ratio *R*_cover_ is expressed as:

(1) $$R_{\mathrm{cover}}=\frac{l_{\mathrm{cover}}}{l}$$

where *l* denotes the total length of the plate, and *l*_cover_ represents the length at which the particle volume fraction on the flat surface exceeds 40% when the burial process is completed.

During the process of movement, the plate is affected by the viscous force and pressure of the fluid. Accordingly, the instantaneous force acting on the plate during motion can be expressed as [44]:

(2) $$\left\{\begin{array}{l} F_{x}=\int_{s}(p_{i}n_{xi}-\tau_{xi}n_{xi})ds\\ F_{y}=\int_{s}(p_{i}n_{yi}-\tau_{yi}n_{yi})ds \end{array}\right.$$

where *F_x_* and *F_y_* denote the instantaneous forces acting on the plate surface in the *x*- and *y*-directions, respectively; *p_i_* represents the pressure on the *i*-th surface element of the plate; *τ_xi_* represents the *x*-direction components of the shear force on the *i*-th surface element of the plate; *τ_yi_* represents the *y*-direction components of the shear force on the *i*-th surface element; and *n_xi_* and *n_yi_* denote the *x*- and *y*-direction components of the unit normal vector of the *i*-th surface element, respectively.

The input power is defined as the time integral of the power output from the plate to the fluid over the entire burial process. It reflects the energy expenditure required for the fish to complete the burial motion: the lower the input power, the less energy is needed. The instantaneous input power of the plate movement during the burial process, *P_t_*, can be expressed as:

(3) $$P_{t}=\int_{s}\left[(p_{i}n_{xi}-\tau_{xi}n_{xi})v_{x}+(p_{i}n_{yi}-\tau_{yi}n_{yi})v_{y}\right]ds$$

where *v_x_* and *v_y_* denote the instantaneous velocities of the *i*-th surface element of the plate in the *x*- and *y*-directions, respectively.

During the entire flapping process of the pectoral fin, the input power *P* is defined as:

(4) $$P=\int_{t}P_{t}dt$$

The burial efficiency *η* is defined as the ratio of the coverage length *l*_cover_ to the input power *P*. The burial efficiency reflects the efficiency of fish in completing the burial action. A higher burial efficiency indicates that the fish can achieve a longer coverage length with the same energy expenditure, or achieve the same coverage area with lower input power. The burial efficiency *η* can be expressed as:

(5) $$\eta =\frac{l_{\mathrm{cover}}}{P}$$

## 3. Numerical Method and Validation

### 3.1. Fluid Solver

The sediment volume fraction at the seabed typically exceeds 40%, which can be approximated as a continuous medium. Therefore, the sediment is defined as a continuum, and the Euler–Euler model is employed to describe the mechanical interactions between the fluid and particles. In the Euler–Euler framework, the interpenetrating multiphase flow is characterized using phase volume fractions, which represent the spatial occupancy of each phase. Each phase satisfies both the continuity and momentum equations. The continuity and momentum equations for phase *q* in the Euler–Euler model are expressed as follows [45]:

(6) $$\begin{array}{l} \frac{\partial }{\partial t}\left(\alpha_{q}\rho_{q}\right)+\nabla \cdot \left(\alpha_{q}\rho_{q}v_{q}\right)={\dot{m}}_{pq}\\ \frac{\partial }{\partial t}\left(\alpha_{q}\rho_{q}v_{q}\right)+\nabla \cdot \left(\alpha_{q}\rho_{q}v_{q}v_{q}\right)=-\alpha_{q}\nabla p+\nabla \cdot \left(\alpha_{q}\tau_{q}\right)+R_{pq}+G_{q}+F_{q}^{int} \end{array}$$

where *α_q_* is the volume fraction of phase *q*, and the sum of the volume fractions of phases *q* and *p* equals 1; *ρ_q_* is the density; *v_q_* is the velocity vector; ${\dot{m}}_{pq}$ denotes the mass source term between phases *p* and *q*; *τ_q_* represents the Newtonian fluid stress tensor; **R***_pq_* is the interphase interaction force between phases *p* and *q*; $F_{q}^{int}$ denotes the internal force of phase *q*; and *G_q_* represents gravity.

The SST *k*-*ω* turbulence model is adopted in combination with the multiphase turbulence interaction model proposed by Simonin et al. The SST *k*-*ω* model belongs to the two-equation model framework of the Reynolds-averaged Navier–Stokes (RANS) equations [46]. It retains the accuracy of the standard *k*-*ω* model in the near-wall region, while providing the numerical stability of the *k*-*ε* model in the free-stream region, thereby combining the advantages of the two types of models. Meanwhile, Simonin’s multiphase turbulence interaction model introduces bidirectional coupling corrections generated by the particle phase into the turbulence equations of the flow field, allowing the feedback effect of particles on turbulence characteristics to be captured. By combining the two, the complex flow features around the fish body and the interaction processes between the fluid and particles can be more accurately described, thus meeting the accuracy requirements of the present numerical simulations [47,48].

The turbulence kinetic energy *k* and specific dissipation rate *ω* in the SST *k*-*ω* model are governed by the following equations:

(7) $$\begin{array}{c} \frac{\partial }{\partial t}\left(\rho k\right)+\frac{\partial }{\partial x_{i}}\left(\rho ku_{i}\right)=\frac{\partial }{\partial x_{j}}\left(\Gamma_{k}\frac{\partial k}{\partial x_{j}}\right)+G_{k}-Y_{k}+S_{k}\\ \frac{\partial }{\partial t}\left(\rho \omega \right)+\frac{\partial }{\partial x_{i}}\left(\rho \omega u_{i}\right)=\frac{\partial }{\partial x_{j}}\left(\Gamma_{\omega }\frac{\partial \omega }{\partial x_{j}}\right)+G_{\omega }-Y_{\omega }+S_{\omega }+D_{\omega } \end{array}$$

where *u_i_* denotes the velocity component; Γ*_k_* and Γ*_ω_* are the effective diffusivities of *k* and *ω*, respectively; *Y_k_* and *Y_ω_* represent the dissipation of *k* and *ω* due to turbulence; *G_k_* and *G_ω_* are the production terms of *k* and *ω*, indicating the generation of turbulence kinetic energy induced by the mean velocity gradient; *S_k_* and *S_ω_* are the particle–turbulence coupling terms introduced by Simonin’s multiphase turbulence interaction model; and *D_ω_* is the cross-diffusion term, which constitutes an important modification distinguishing the SST *k-ω* model from the standard *k-ω* model, as it suppresses the excessive growth of the specific dissipation rate *ω* in the free-stream region and enhances the accuracy of shear flow computations.

The Gidaspow interphase drag model [49] is employed to describe the momentum coupling between the fluid phase and the particle phase. In dilute particle regions, the Wen-Yu drag model [49] is adopted, while in dense particle regions, the Ergun drag model [49] is used. This unified treatment under varying volume fraction conditions accounts for both dilute and dense regimes, making it suitable for characterizing particle transport behavior during fish burying processes.

The Phase Coupled SIMPLE (PC-SIMPLE) algorithm [50] is applied to solve the pressure-velocity coupling equations. The PC-SIMPLE method simultaneously corrects the pressure field and couples the momentum exchange and volume fraction variations among phases in each iteration, thereby ensuring improved convergence efficiency and stability for multiphase flows with high volume fractions. For pressure interpolation, the Pressure Staggering Option (PRESTO!) scheme is employed, which, compared with conventional interpolation methods, ensures higher accuracy in pressure field computations and effectively suppresses numerical oscillations when dealing with complex flows involving strong accelerations, vortex structures, and non-orthogonal grids. In the selection of discrete schemes, the momentum equations, turbulence kinetic energy, and specific dissipation rate are solved using the second-order upwind scheme, while the volume fraction adopts the Quadratic Upstream Interpolation for Convective Kinematics (QUICK) scheme.

**Figure 2:**
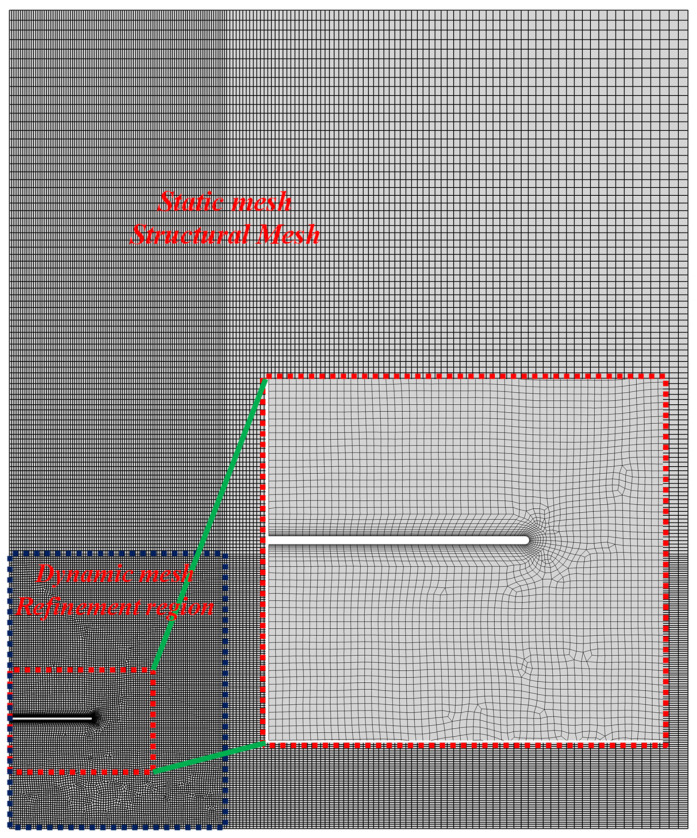
Computational mesh used in this study.

ANSYS Fluent 2024 is used to solve the above numerical calculation model. Figure 2 presents the computational mesh employed in this study. The mesh consists of both static and dynamic grids, with the static mesh completely composed of the structure grids, while the dynamic mesh is mainly composed of the structure grids. The boundary displacement is prescribed using Fluent’s User Defined Function (UDF) capability. The dynamic mesh employs the Radial Basis Function (RBF) method. The RBF method interpolates the boundary displacement to the whole computational domain by interpolation method, which is well-suited for handling mesh distortion induced by large boundary deformations [51].

According to the observations of Seamone et al. on the self-burial process of rays, the fish remain stationary on the sediment bed after completing the pectoral fin motion, during which the particles stirred up by the fin gradually settle onto the body surface under the action of gravity. Therefore, the initialization stage of the numerical simulation is completed before 0.1 s, and then the flapping/burial process of the plate is realized in the given boundary position in the UDF. After the plate flapping is completed, the particles are allowed to settle freely under gravity, and the free-fall time of the particles is 3 s. Therefore, the total simulation time (in seconds) for each case is given by 0.1 + *N*/*f* + 3.

### 3.2. Validations

The accuracy of the numerical simulation algorithm in this paper is verified through a case of water flow impacting a sand bed. This case is based on Aderibigbe et al. [52]. In this validation case, the water jet nozzle was fixed at the top of the tank and just submerged in water. The bottom of the tank was uniformly covered with sand. Under the impingement of the water jet, a scour pit was formed in the sandbed. By comparing the shape of the scour hole between the experimental results and the numerical simulation results, the accuracy of the proposed numerical method was verified.

**Figure 3:**
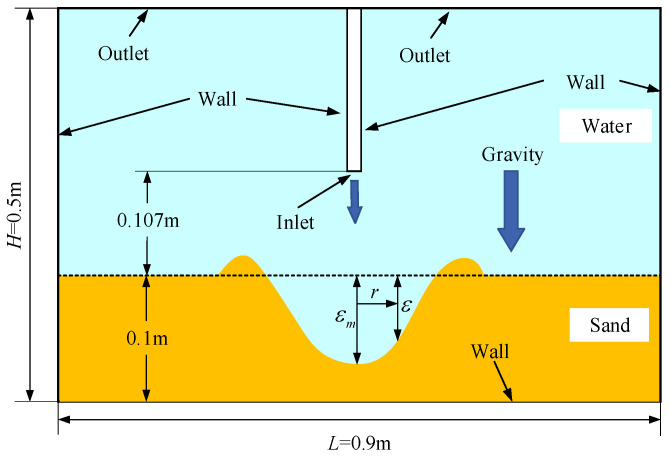
Schematic illustration of water flow impinging on a sand bed.

As illustrated in Figure 3, the computational domain has a length of 0.9 m and a height of 0.5 m. The particle diameter is 2 mm, and the particle density is 2650 kg/m^3^. The nozzle diameter is 2.5 mm, and the initial water velocity is 3.1 m/s. At the initial moment, the nozzle is positioned 107 mm above the sandbed, which has a thickness of 0.1 m and a particle volume fraction of 60%. After the scouring process, the scour pit radius is denoted by *r*, the scour pit depth by *ε*, and the maximum scour pit depth by *ε_m_*.

**Figure 4:**
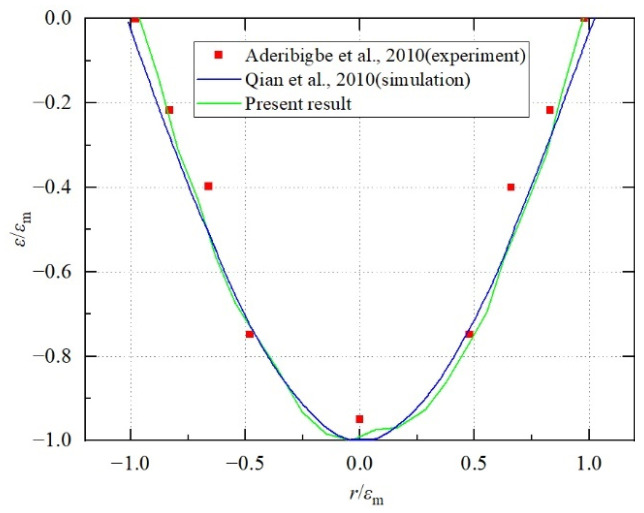
Comparison of scour pit morphologies under water flow impingement on a sand bed [52,53].

The results of the sand pit obtained by the numerical model in this paper are shown in Figure 4. It can be observed that the results of this study are in good agreement with the numerical simulation results of Qian et al. [53] and the experimental results of Aderibigbe et al. [52], thereby validating the accuracy of the proposed numerical method.

**Figure 5:**
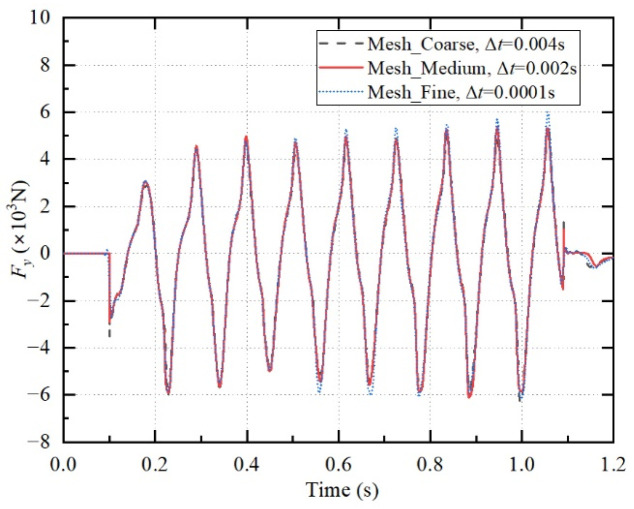
Comparison of the *y*-direction force among the three cases. The plate length is *l* = 15 cm, the plate thickness *h* = 5 mm, the particle diameter *d*_p_ = 0.9 mm, the flapping number *N* = 10, the flapping frequency *f* = 9 Hz, and the maximum bending angle of the plate *θ*_max_ = 140°.

To ensure the independence of the present numerical calculation model from both time step size and grid resolution, three different grid sizes and various time steps were used to verify the cases. The detailed settings of the three cases are summarized in Table 2: for the coarse mesh, the first boundary-layer grid size is 0.45 mm, the refined-region grid size is 6 mm, the number of grids is 22,000, and the time step is 0.0003 s; for the medium mesh, the first boundary-layer grid size is 0.3 mm, the refined-region grid size is 4 mm, the number of grids is 40,000, and the time step is 0.0002 s; for the fine mesh, the first boundary-layer grid size is 0.2 mm, the refined-region grid size is 3 mm, the number of grids is 77,000, and the time step is 0.0001 s. By comparing the *y*-direction force *F_y_* of the plate among the three cases, the independence of the numerical calculation model from time step and grid resolution was validated. The calculation results of the three examples are shown in Figure 5. The medium mesh, combined with a time step of 0.0002 s, provided sufficiently accurate results. Therefore, all subsequent numerical simulations employed the medium mesh and a time step of 0.0002 s.

## 4. Orthogonal Experimental Scheme

In this study, an orthogonal experimental design was adopted to evaluate the effects of plate length, flapping frequency, flapping number, flapping amplitude, and particle size on the self-burial performance. These five factors are mutually independent, and each includes multiple levels. If the traditional control variable method were applied, the number of tests would be enormous, and it would remain difficult to identify the relative importance of the factors. In contrast, the orthogonal design allows the simultaneous investigation of multi-factor and multi-level effects on self-burial behavior within a limited number of trials, while effectively distinguishing the primary and secondary influences of the factors. In this paper, the burial ratio, input power, and burial efficiency were selected as the evaluation indices of self-burial performance, and the significant differences in factors on the three evaluation indices are emphatically analyzed.

Specifically, five factors were defined in the experimental design (see Table 3): Factor 1, fish body length (3, 6, 9, 12, and 15 cm); Factor 2, flapping frequency (5, 6, 7, 8, and 9 Hz); Factor 3, flapping number (2, 4, 6, 8, and 10 times); Factor 4, flapping amplitude (60°, 80°, 100°, 120°, and 140°); and Factor 5, sand particle size (0.1, 0.3, 0.5, 0.7, and 0.9 mm). Based on the principles of orthogonal design, an experimental scheme comprising 25 groups of representative combinations was constructed (see Table 4). This scheme ensured a balanced distribution of all factors and levels within the test sequence. For each group of experiments, the burial ratio, input power, and burial efficiency were calculated, and then the contribution rate of each factor was quantified by range analysis and variance analysis to clarify the primary and secondary influence order of different factors on the self-burial effect.

## 5. Results

### 5.1. Orthogonal Experiment Results

Table 5 lists the coverage ratio, coverage size, input power, and burial efficiency results of each test group. In the range analysis of the orthogonal experiments, for a given factor at a specific level, the results corresponding to that level are summed to obtain the *K* value of the factor at that level. On this basis, the *K* value is divided by the average number of experimental repetitions *r* at the corresponding level to yield the average value at the current level *K*_avg_, that is:

(8) $$K_{\mathrm{avg}}=\frac{K}{n}$$

where *n* represents the number of experimental repetitions at this level.

By comparing the *K* values of different factors at various levels, one can determine the relative advantages or disadvantages of each level. The range *R* is defined as the difference between the maximum and minimum *K* values of a certain factor. A larger value of *R* indicates that the factor has a more significant influence on the experimental results.

In the orthogonal experimental design of this study, the levels of each factor were not completely consistent. For instance, when the fish body length is 3 cm, the effective burial effect is not produced, and the coverage ratio remains 0. Therefore, Factor 1 (fish body length) has four valid levels, whereas other factors have five valid levels. This different setting of the level number constitutes a mixed orthogonal table. In range analysis, this discrepancy in the number of levels affects the evaluation of factor influence and must be addressed in combination with the conversion method.

To overcome this issue, the range values need to be converted. The convert coefficient *d* is determined by the number of levels for each factor, and its value can be obtained from the coefficient table shown in Table 6 (for four levels, *d* = 0.45; for five levels, *d* = 0.40). The corrected range value *R*′ can be expressed as:

(9) $$R^{\prime }=d\cdot R\cdot \sqrt{r}$$

where *R* denotes the original range value, *d* is the conversion coefficient, and *r* represents the average number of repetitions for each level under this factor.

By introducing the conversion coefficient and recalculating the range value *R*′, it can eliminate the influence of inconsistency in the number of factor levels (e.g., the 3 mm fish body length producing no burial effect). Therefore, in the range analysis of this study, the converted range value *R*′, rather than the original range *R*, is adopted as the basis for evaluating the influence strength of each factor. This guarantees the reliability of assessing the factors of fish body length, flapping frequency, flapping number, flapping amplitude, and sand grain size on coverage ratio, input power, and burying efficiency.

**Figure 6:**
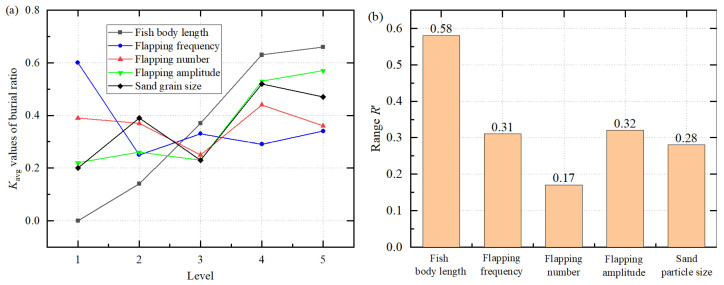
(**a**) Average value *K*_avg_ of the burial ratio at different levels, and (**b**) range value *R*′ of different factors.

According to the range analysis results of the coverage ratio presented in Table 7 and Figure 6, significant differences exist in the influence of different factors on the coverage ratio. The ranking of the converted range values *R*′ is as follows: fish body length (0.58) > flapping amplitude (0.32) > flapping frequency (0.31) > sand grain size (0.28) > flapping number (0.17). Among them, the range of fish body length is the largest, indicating that it exerts the most significant influence on coverage ratio and serves as the primary controlling factor. Flapping amplitude and frequency follow, while the effects of sand grain size and flapping number are relatively limited.

Based on the analysis of the *K*_avg_ values, the optimal combination for achieving maximum coverage ratio is: fish body length of 15 mm, flapping frequency of 5 Hz, flapping number of 8, flapping amplitude of 140°, and sand grain size of 0.7 mm. Under these parameter conditions, the coverage ratio reaches its optimum. Further analysis reveals that the coverage ratio increases markedly with increasing fish body length, suggesting that larger body sizes are most conducive to the self-burial process.

Table 8 presents the results of the multifactor Analysis of Variance (ANOVA) for the burying ratio. The results show that fish body length, flapping frequency, flapping amplitude, and sand grain size all have significant effects on the coverage ratio (*p* < 0.05), with fish body length and flapping amplitude being the most significant (*p* < 0.01). In contrast, the effect of flapping number is not significant (*p* = 0.157). This indicates that the coverage ratio primarily depends on the geometric scale of the fish body and the flapping amplitude, while flapping frequency and grain size play a moderating role, and the contribution of flapping number is limited.

**Figure 7:**
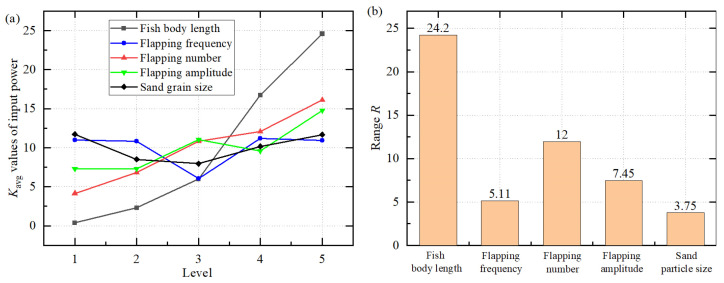
(**a**) Average value *K*_avg_ of the input power at different levels, and (**b**) range value *R* of different factors.

Table 9 and Figure 7 show the range analysis results for input power. According to the ranking of ranges, the order is: fish body length (24.22) > flapping number (11.95) > flapping amplitude (7.45) > flapping frequency (5.11) > sand grain size (3.75). Among these, fish body length has the most significant effect, while flapping number and flapping amplitude also exert considerable influence. The effects of flapping frequency and sand grain size on input power are relatively minor. The optimal parameter level is: fish body length of 3 cm, flapping frequency of 7 Hz, flapping number of 2, flapping amplitude of 60°, and sand grain size of 0.5 mm. Under this combination, the required input power is relatively low. It can be seen that an increase in the length of the fish body will significantly enhance the input power. The reason for this might be that after the fish’s body size is enlarged, the fluid resistance and the range of particle disturbances increase significantly, thus requiring a higher power input.

Table 10 presents the multifactor ANOVA results for input power. It can be seen that fish body length has a significant effect on input power (*p* < 0.05), while the other factors have no significant effect. (*p* > 0.05). This indicates that input power mainly depends on the geometric size of the plate itself, with the remaining kinematic factors and particle characteristics playing secondary roles in energy consumption.

**Figure 8:**
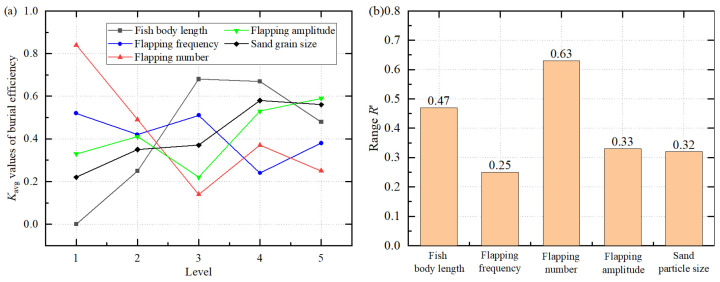
(**a**) Average value *K*_avg_ of the burial efficiency at different levels, and (**b**) range value *R*′ of different factors.

The burying efficiency combines the performance of the coverage ratio and input power, making it a key indicator for evaluating self-burying capacity. Table 11 and Figure 8 show the range analysis results for burying efficiency. According to the ranking of R′, the order is: flapping number (0.63) > fish body length (0.47) > flapping amplitude (0.33) > sand grain size (0.32) > flapping frequency (0.25). Flapping number shows the largest range, indicating that it has the most prominent influence on burying efficiency, followed by fish body length, while flapping frequency has the weakest effect.

The optimal parameter combination is: fish body length of 9 cm, flapping frequency of 5 Hz, flapping number of 2, flapping amplitude of 140°, and sand grain size of 0.7 mm. Under these conditions, coverage effect and energy consumption reach a balance.

Table 12 shows the multifactor ANOVA results for burying efficiency. The results indicate that the influence of each factor on burial efficiency is not significant (*p* > 0.05). This may be since the burial efficiency combines the coverage ratio and the input power, with its significance determined by the joint influence of multiple factors, thereby weakening the significance of single factors. Nevertheless, the range analysis still suggests that flapping number and fish body length play important roles in improving burying efficiency.

Through the orthogonal experiment and range analysis, it is clear that different factors exert distinct effects on coverage ratio, input power, and burying efficiency. For the coverage ratio, fish body length is the dominant factor, with the largest range, indicating that larger body size is favorable for achieving higher self-burial coverage ratios. Flapping amplitude and frequency follow, while flapping number and sand grain size have the weakest effects. The range analysis of input power shows that fish body length remains the key factor influencing energy consumption, with flapping number and flapping amplitude having secondary effects, while flapping frequency and sand grain size exert an insignificant influence. This suggests that input power is mainly determined by fish body size, while other kinematic parameters and particle properties play a limited role in regulating energy consumption. For burying efficiency as a comprehensive indicator, flapping number and fish body length exhibit larger ranges, indicating that increasing body size and flapping number can significantly enhance overall burying efficiency, and the influence of other factors is relatively weak. Multifactor ANOVA further confirms these conclusions: the coverage ratio was mainly significantly influenced by the fish body length and the flapping amplitude, while the input power was significantly dependent on the fish body length. The single-factor significance of the burial efficiency was relatively weak, but through the range analysis, the important roles of the flapping frequency and the fish body length could still be identified.

In summary, the geometric size of the fish body and its kinematic parameters (particularly flapping frequency and amplitude) play central regulatory roles in the self-burial process, and the role of sand grain size is relatively secondary. In the next step, visualization analysis of cloud maps will be conducted to reveal the specific mechanisms through which each factor influences particle deposition, fluid disturbance, and energy consumption, thereby further validating and explaining the regularity observed in the orthogonal experimental results.

### 5.2. Flow Evolution Analysis

**Figure 9:**
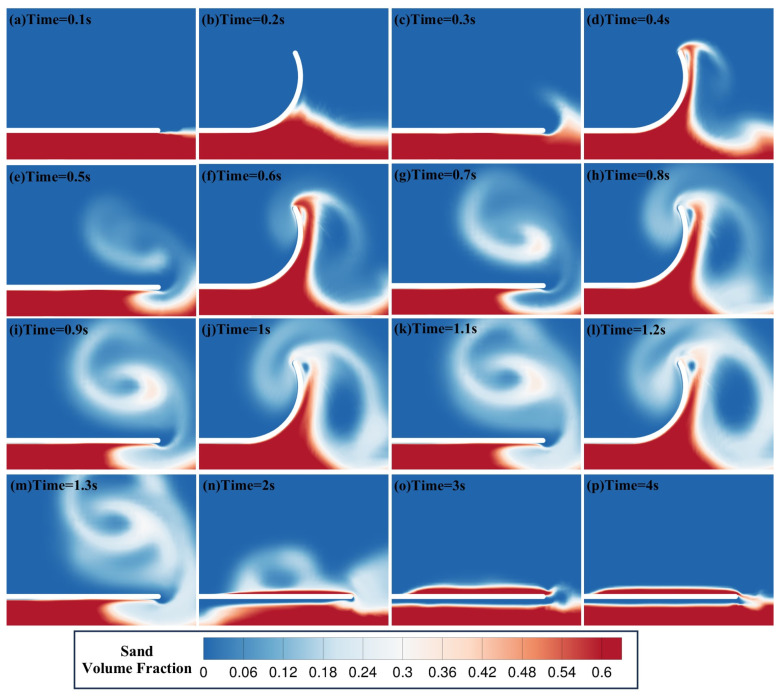
Time evolution of the sand volume fraction during the flapping/burial process of the flat plate in Case 16. Note: (**b**,**d**,**f**,**h**,**j**,**l**) correspond to the instants when the plate reaches its maximum bending angle during each flap.

Benthic fish achieve self-burying behavior in seabed sediments through the flapping of their pectoral fins. To elucidate this biological phenomenon from a numerical simulation perspective, the particle volume fraction contours, vorticity contours, velocity vector fields, and pressure fields of Case 16 were analyzed to examine the particle transport characteristics and fluid motion characteristics induced by fin flapping. Figure 9 illustrates the temporal evolution of the sand volume fraction with time during the flapping-burying process in Case 16. As shown, with the periodic up-and-down flapping of the plate, particles beneath the plate are fluidized and lifted upward, accumulating above the plate and achieving self-burial. In the early stage of self-burial (0.1–0.4 s), particles mainly concentrate near the lower surface of the plate, exhibiting an accumulation pattern. As time progresses (0.5–1.2 s), more particles are entrained by the flapping-induced flow, and particles are rolled up to form a high-concentration particle band that moves toward the upper side of the plate. In the later stage (2–4 s), as flapping ceases, particles gradually deposit on the upper surface of the plate, forming a relatively stable covering layer. Thus, the flapping process significantly alters the stability of particle accumulation in the flow field, enabling particle fluidization, transport, and deposition.

**Figure 10:**
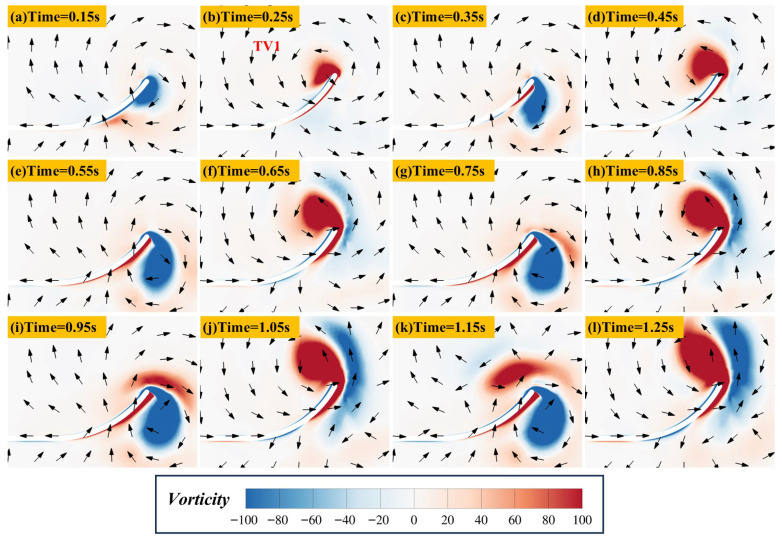
Time evolution of the vorticity and velocity vectors during the flapping/burial process of the flat plate in Case 16. Note: (**a**,**c**,**e**,**g**,**i**,**k**) correspond to the midpoints of the upward flapping motions, (**b**,**d**,**f**,**h**,**j**,**l**) correspond to the midpoints of the downward flapping motions.

Figure 10 shows the vorticity and velocity vector distributions during the flapping-burying process in Case 16. It can be observed that strong shear layer separation occurs at the plate edges during each upward and downward flapping motion, inducing vortex structures at the plate tips. These vortex structures resemble the wingtip vortices of birds and aircraft. Their formation can be explained as follows: during upward flapping, the flow velocity below the plate decreases, leading to higher pressure, while the velocity above the plate increases, resulting in lower pressure. High-pressure and low-pressure fluids meet at the end of the plate, and the high-pressure fluid flows towards the upper part of the plate, generating wingtip vortices. During downward flapping, the vortex direction reverses. As the plate moves upward, fluid is transported from beneath the plate to above it. The alternating appearance of wingtip vortices during the flapping cycle induces a periodic entrainment–deposition process of the particle. Therefore, the unsteady flow distribution induced by flapping serves as the core driving force for particle fluidization and deposition.

**Figure 11:**
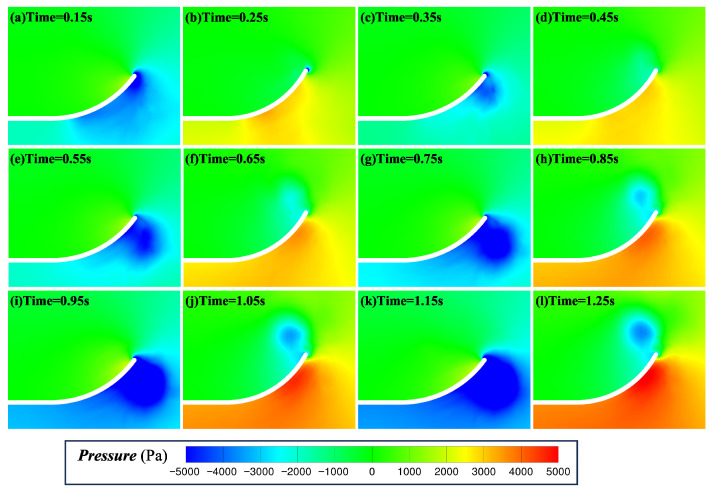
Time evolution of the pressure during the flapping/burial process of the flat plate in Case 16. Note: (**a**,**c**,**e**,**g**,**i**,**k**) correspond to the midpoints of the upward flapping motions, (**b**,**d**,**f**,**h**,**j**,**l**) correspond to the midpoints of the downward flapping motions.

Figure 11 presents the temporal evolution of pressure during the flapping-burying process in Case 16. A significant pressure difference exists between the upper and lower surfaces of the plate during its motion. In upward flapping, a low-pressure region forms beneath the plate, and the fluid continuously entrains the particles at the bottom. In downward flapping, a high-pressure region develops beneath the plate, continuously transporting fluidized particles toward the upper surface. This periodic pressure distribution drives the surrounding fluid, continuously entraining particles from the bottom to the upper surface. The alternating pressure fields on both sides of the plate during flapping facilitate the uniform deposition of particles on the upper surface. Thus, the periodic variation in the pressure field provides a critical driving mechanism for particle transport.

**Figure 12:**
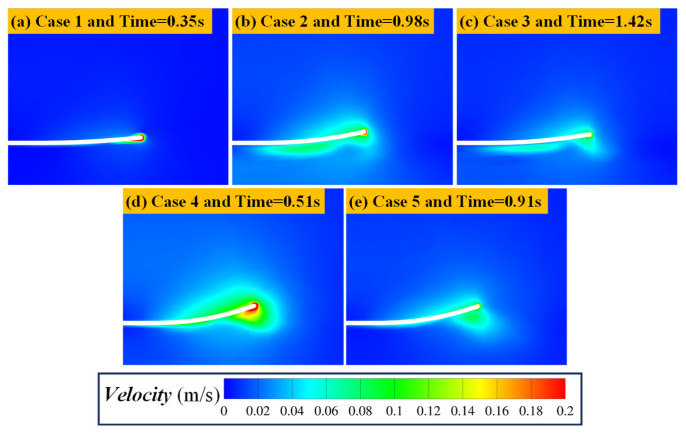
Flow field velocity distribution of the five 3-cm flat plate cases (Case 1, Case 2, Case 3, Case 4, and Case 5) at the moment when the velocity beneath the lower surface reaches its maximum. This instant approximately corresponds to the mid-upstroke of the plate, with the time given by 0.1 + (*N* − 0.75)/*f*.

**Figure 13:**
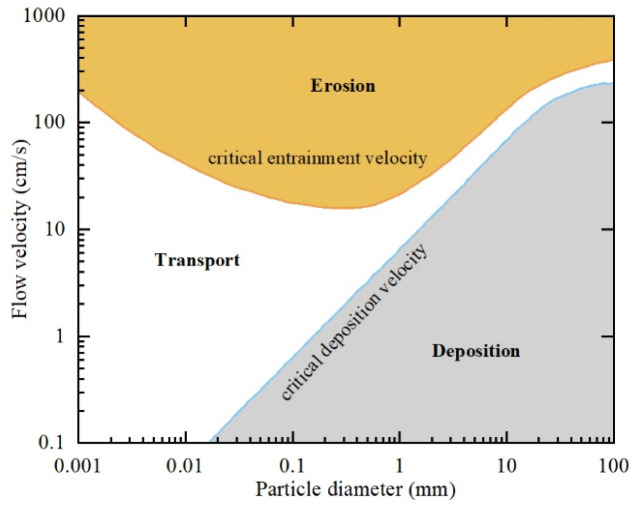
Relationship between flow velocity and the motion states of particles with different grain sizes (erosion, transport, and deposition) [54].

Figure 12 illustrates the velocity distribution at the moment of maximum velocity on the lower surface of the plate for five 3 cm plate cases (Case 1–Case 5). This moment approximately corresponds to the mid-phase of upward motion, at time 0.1 + (*N* − 0.75)/*f*. The body length of the five groups of cases was 3 cm, and all failed to produce effective burial behavior. The Hjulström curve can be used to describe the motion of particles in water. The Hjulström curve [54] (Figure 13) is an empirical diagram of the motion behavior of granular materials in water, which is used to quantitatively describe the relationship between water flow velocity and the motion states of particles of different sizes (erosion, transport, and deposition). According to this curve, the motion state of particles is divided into three categories: (1) When fluid velocity is below the critical start velocity, particles remain at rest. (2) When fluid velocity exceeds the critical start velocity, the force of the fluid on the particles exceeds the gravity and interparticle cohesion, leading to erosion, once the fluid rolls up the particles, only a small flow rate is needed to maintain the motion state of the particles. At this time, the particles are in the transportation stage. (3) When the flow velocity decreases below the critical deposition velocity, particles settle as the fluid force becomes insufficient to counter gravity and interparticle cohesion. Importantly, the critical start velocity varies with particle diameter. For the particle diameter (0.1–0.9 mm) in this study, the corresponding critical starting velocity is 0.2–0.35 m/s. Combined with the velocity distribution results in Figure 12, it can be seen that the fastest velocity on the lower surface of the plate is approximately 0.1 m/s. At this time, the short plate has limited disturbance to the flow field during the flapping process. The velocity on the lower surface of the plate is low and lower than the critical starting velocity of the particles. Although the particles are disturbed by the fluid, they are difficult to fluidize and transport, so they fail to produce burial behavior.

**Figure 14:**
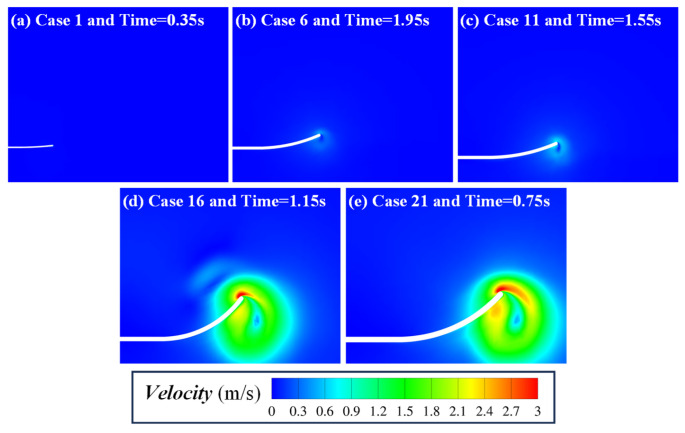
Flow field velocity distribution of the flat plate cases (Case 1, Case 2, Case 3, Case 4, and Case 5) at the moment when the velocity beneath the lower surface reaches its maximum. This instant approximately corresponds to the mid-upstroke of the plate, with the time given by 0.1 + (*N* − 0.75)/*f*.

To confirm that the larger fish size may be beneficial to the coverage and deposition of particles from the perspective of numerical simulation, Figure 14 shows the flow field velocity distributions beneath the plate at the moment of maximum velocity for different plate lengths (Case 1, Case 6, Case 11, Case 16, and Case 21). The results indicate that as plate length increases, the intensity and spatial influence range of the generated velocity field by flapping grow significantly, making it easier for local velocities to exceed the critical start velocity defined by the Hjulström curve. Consequently, particles become fluidized and burial occurs. This explains why longer plates produce clear burial effects, while shorter plates fail to do so.

In summary, by integrating the Hjulström curve with flow field analyses across different cases, the inability of shorter plates to induce burial and the effectiveness of longer plates in achieving burial can be clearly explained. However, it should be pointed out that flow field contours alone cannot directly reveal the relative effects of motion parameters (flapping amplitude, frequency, and number) and environmental parameters (particle size) on input power. Similarly, it is difficult to intuitively analyze the influence of various factors on the burial efficiency from the flow field cloud diagram. At the macroscopic level, burying efficiency is jointly influenced by fish body length, flapping amplitude, frequency, number, and particle size. At the microscopic level, it is affected by complex interactions involving fluid vorticity, pressure distribution, and interparticle forces. Thus, burying efficiency exhibits multifactor coupling effects, and relying solely on flow field contours cannot accurately uncover its underlying mechanisms. Therefore, subsequent research should adopt the method of controlled variables to control and compare key parameters, thereby elucidating the mechanisms by which different factors influence fish burying behavior.

## 6. Discussion

The orthogonal experiments and range analysis results indicate that fish body length has the most significant influence on the coverage ratio. Longer bodies can generate larger-scale fluid disturbances during flapping, causing more sediment particles to deposit over the body surface and thus increasing the self-burial coverage ratio. However, this pattern contradicts the observations of Corn et al. [40] on the self-burial of the *Parophrys vetulus*. Corn et al. reported that body size had no significant effect on burial ratio, with both large and small individuals achieving comparable burial extents. The reason for this difference is likely to be related to the flapping frequency. According to their observations, larger fish bury themselves at lower flapping frequencies, whereas smaller fish employ higher flapping frequencies. Therefore, it can be inferred that when large and small fish bury at the same flapping frequency, the coverage ratio of larger fish will be significantly higher than that of smaller ones. This inference not only validates the significant influence of body length on coverage ratio but also aligns with the conclusions of the present orthogonal experiments. The numerical simulation results further corroborate this finding: as plate length increases, the velocity field intensity and disturbance range generated by flapping are substantially enhanced, making it easier for local flow velocity to exceed the critical start threshold defined by the Hjulström curve, thereby fluidizing particles and initiating the burial process.

The orthogonal experiments and range analysis also demonstrate that particle size exerts the weakest effect on coverage ratio. The reason for this rule can be explained in three aspects. First, in terms of body structure, the eyes of many benthic fish are located on the back of the body, that is, facing the water surface and back to the seabed, which may lead to the inability to perceive the particle size information of seabed sediments. Thus, burial behavior may not rely on the fish’s active selection of granular sediments of specific sizes. Second, from the perspective of biological evolution, burial is an adaptive strategy for predator avoidance and prey capture. To achieve self-burial across diverse benthic environments, benthic fish must be capable of burying themselves in sediments of varying sizes rather than depending on a specific grain size. Third, from the perspective of the present numerical simulations, the Hjulström curve (Figure 13) shows that for particles with diameters between 0.1 mm and 0.9 mm, the critical start velocity ranges from 0.2 m/s to 0.35 m/s. The critical start velocity difference in different particle sizes is much lower than the flow velocity difference caused by the change in kinematic parameters. As shown in the flow velocity field contours in Figure 14, when the velocity beneath the plate reaches its maximum, the bottom-surface velocity in Case 21 is nearly ten times that in Case 1. Compared with the variation in critical start velocity caused by particle size, the effect of fish body motion parameters on flow field velocity intensity is far more pronounced. Therefore, while sediment grain size does affect coverage ratio to some degree, its role is much weaker than the fluid disturbances generated by the geometric parameters and kinematic parameters of the fish body.

Regarding input power, it shows a strong correlation with fish body length. This result means that energy consumption is more determined by geometric scale. This phenomenon is also reflected in nature: large *Potamotrygon motoro*, for example, generate intense vortices through wide-amplitude pectoral fin flapping during burial. However, their overall flapping frequency remains low, and the burial process takes longer, thereby reducing energy expenditure per unit time [40].

Flapping amplitude and frequency emerged as secondary factors in this study, but they still contribute to self-burial by modulating the intensity of fluid disturbance. Moderate increases in amplitude and frequency enhance fluidization beneath the body and promote particle transport onto the dorsal surface, thus improving the coverage ratio. However, excessively high frequencies may reduce burial efficiency. This echoes the experimental findings of McKee et al. [43]: although a silicone biomimetic model achieved greater coverage areas at frequencies above 10 Hz, the excessively high frequencies far exceeded the physiological capacity of natural fish, suggesting that benthic fish operate within adaptive boundaries of kinematic parameters.

From an engineering standpoint, the findings of this study offer important reference value for the benthic residence method of bionic self-buried UUV. First, the size of flapping wings dictates both the scale and intensity of fluid disturbances. Wings that are too small may fail to generate sufficient disturbance to exceed the critical start velocity, leading to burial failure, and too large wings increase structural load and input energy demand. This indicates that when designing self-buried UUVs, a balance must be struck between airfoil parameters and burial capacity. Second, strategies for coordinating kinematic parameters can be directly applied in biomimetic design. For instance, larger fish typically achieve burial at lower flapping frequencies, while smaller fish do so at higher frequencies. By combining geometric and kinematic parameters appropriately, it is possible to maintain high coverage ratios while reducing maximum instantaneous energy consumption, thereby extending endurance. Finally, the weak influence of sediment grain size suggests that biomimetic self-burial UUVs possess strong adaptability across diverse seabed environments, without requiring parameter adjustments for different sediments.

Although this study investigated the influence of geometric and kinematic parameters of benthic fish on self-burial behavior through numerical simulation and orthogonal experiments, there are still certain limitations. First, the simulations did not account for pectoral fin flexibility; instead, they directly specified fin boundary displacements. Flexible deformation of the fins may play an important role in self-burial. Second, the orthogonal experiments used here only allow for evaluating the influence of different factors. Future work could employ controlled variable methods to reveal the influence mechanisms of each factor on self-burial performance.

## 7. Conclusions

Based on the numerical simulation method, this study established a two-dimensional fluid-particle coupling model for the burial behavior of benthic fish. The pectoral fins are simplified as flexible plates, and the entire self-burial process induced by fin flapping is reproduced using an Euler–Euler multiphase flow framework, the SST *k–ω* turbulence model, and the Gidaspow interphase drag model. The spatiotemporal evolution of particle volume fraction, fluid vorticity, velocity vectors, and pressure fields during burial was analyzed. Within the orthogonal experimental framework, the relative influence of body length, flapping frequency, flapping number, flapping amplitude, and sediment grain size on the self-burial effect was systematically examined. Three quantitative indices—coverage ratio, input power, and burial efficiency—were employed for evaluation. Based on the numerical simulation and orthogonal experimental results, the following conclusions can be drawn:

(1) Orthogonal analysis reveals that fish body length is the most significant factor affecting both burial coverage ratio and input power. Larger body lengths generate stronger vortical structures and wider fluid disturbances, thereby markedly increasing coverage ratio. However, although larger individuals are more capable of achieving burial, this comes at the cost of greater energy consumption; smaller individuals, while requiring less energy, are less effective in producing sufficient sediment coverage. Flapping amplitude and frequency also play important roles. The higher flapping frequency contributes to the formation of a high-intensity vortex and the acceleration of particle suspension in a short time; however, if the frequency is too high, the burial efficiency may decrease due to the inertial effect of the fluid. Sediment grain size has a certain influence on burial performance, but compared to the geometric and kinematic parameters of the fish body, its role is relatively secondary.
(2) The numerical simulations visualize the evolution of the flow field during the self-burial process driven by pectoral fin flapping. The burial process of benthic fish can be described as follows: periodic flapping of the fins generates a vortex structure at the end of the wingtip vortex, which alternately acts on the flow field, entraining and transporting sediment upward until it gradually deposits on the dorsal surface of the plate to form a coverage layer. Meanwhile, alternating low-pressure suction and high-pressure thrust develop above and below the surface of the plate, enhancing sediment entrainment and transport. When the local flow velocity exceeds the critical start threshold of sediment particles, the sediments are effectively fluidized and enter a transport state, ensuring the continuity of the burial process. The periodic disturbance of the velocity field provides a stable transport pathway, maintaining a circulation loop from sediment rolling up to transportation.

This study elucidates the mechanisms by which geometric and kinematic parameters influence the self-burial process of benthic fish, offering important implications for the design of biomimetic self-burial UUVs. First, wing surface size directly determines the scale and intensity of fluid disturbances: undersized wings may fail to exceed the critical start velocity, leading to burial failure, while oversized wings substantially increase structural loads and energy consumption. Therefore, a balance between burial capacity and energy efficiency is essential. Second, kinematic parameters should be coordinated with geometric scale: larger wingspan may adopt lower flapping frequencies, amplitudes, and numbers to reduce instantaneous power requirements, whereas smaller spans require higher-frequency and larger-amplitude flapping to maintain sufficient coverage. This synergistic relationship between wing geometry and motion kinematics can guide the optimization of motion control strategies for self-burial UUVs. Finally, the influence of sediment particle size on the burial performance is limited. In comparison, the impact of kinematic parameters and geometric parameters on the burial performance is more significant. Therefore, more attention should be paid to the reasonable selection and optimization of motion parameters and geometric parameters in the design of self-buried UUVs.

## Figures and Tables

**Table 1:** Parameters in numerical calculation models.

Symbol	Value	Description
*L*	1.25 m	length of the computational domain
*H*	1.5 m	height of the computational domain
_——_	0.2 m	height of particle domain
*l*	3 cm, 6 cm, 9 cm, 12 cm, 15 cm	length of the plate
*h*	1 mm, 2 mm, 3 mm, 4 mm, 5 mm	thickness of the plate
*ρ*_f_	998.2 kg/m^3^	fluid density
*v*_f_	1.003 × 10^−3^ kg/(m·s)	dynamic viscosity of fluid
*ρ*_p_	2500 kg/m^3^	particle density
*v*_p_	20 kg/(m·s)	dynamic viscosity of particles
*d*_p_	0.1 mm, 0.3 mm, 0.5 mm, 0.7 mm, 0.9 mm	diameter of particles
*θ*_p-initial_	63%	initial volume fraction of the particle region
*N*	2, 4, 6, 8, 10	flapping number
*f*	5 Hz, 6 Hz, 7 Hz, 8 Hz, 9 Hz	flapping frequency
*θ*_max_	60°, 80°, 100°, 120°, 140°	maximum bending angle of the plate

**Table 2:** Configuration details of the three computational cases.

Category	Time Step	First Boundary-Layer Grid Size	Number of Grids	Refined-Region Grid Size
Mesh_Coarse	0.0003 s	0.45 mm	22,000	6 mm
Mesh_Medium	0.0002 s	0.3 mm	40,000	4 mm
Mesh_Fine	0.0001 s	0.2 mm	77,000	3 mm

**Table 3:** Parameters for Orthogonal Experiment.

		Level 1	Level 2	Level 3	Level 4	Level 5
Factor 1	Fish body length/cm	3	6	9	12	15
Factor 2	Flapping frequency/Hz	5	6	7	8	9
Factor 3	Flapping number	2	4	6	8	10
Factor 4	Flapping amplitude/°	60	80	100	120	140
Factor 5	Sand particle size/mm	0.1	0.3	0.5	0.7	0.9

**Table 4:** Orthogonal Experimental Table.

No.	Factor 1	Factor 2	Factor 3	Factor 4	Factor 5	No.	Factor 1	Factor 2	Factor 3	Factor 4	Factor 5
1	1	1	1	1	1	14	3	4	5	1	2
2	1	2	3	4	5	15	3	5	2	4	1
3	1	3	5	2	4	16	4	1	3	5	2
4	1	4	2	5	3	17	4	2	5	3	1
5	1	5	4	3	2	18	4	3	2	1	5
6	2	1	5	4	3	19	4	4	4	4	4
7	2	2	2	2	2	20	4	5	1	2	3
8	2	3	4	5	1	21	5	1	2	3	4
9	2	4	1	3	5	22	5	2	4	1	3
10	2	5	3	1	4	23	5	3	1	4	2
11	3	1	4	2	5	24	5	4	3	2	1
12	3	2	1	5	4	25	5	5	5	5	5
13	3	3	3	3	3						

**Table 5:** Burial ratio, burial size, input power, and burial efficiency for each experimental group.

No.	Burial Ratio	Burial Size (cm)	Input Power (MW)	Burial Efficiency (cm/MW)
1	0	0	0.08	0
2	0	0	0.45	0
3	0	0	0.49	0
4	0	0	0.35	0
5	0	0	0.49	0
6	0.45	2.69	4.52	0.6
7	0	0	1.32	0
8	0.2	1.17	4.05	0.29
9	0.05	0.3	0.81	0.37
10	0	0	0.81	0
11	0.68	6.08	7.26	0.84
12	0.7	6.26	3.48	1.8
13	0	0	7.45	0
14	0.14	1.26	6.23	0.2
15	0.34	3.07	5.48	0.56
16	0.97	11.6	22.28	0.52
17	0.2	2.42	25.66	0.09
18	0.63	7.6	6.19	1.23
19	1	12	25.22	0.48
20	0.36	4.33	4.24	1.02
21	0.88	13.16	20.73	0.64
22	0.33	5	23.23	0.22
23	0.84	12.55	12.14	1.03
24	0.27	4.04	23.26	0.17
25	1	15	43.61	0.34

**Table 6:** Conversion coefficient table.

Number of Levels	4	5
Convert coefficient *d*	0.45	0.4

**Table 7:** Results of the range analysis of the burial ratio.

Item	Level	Fish Body Length	Flapping Frequency	Flapping Number	Flapping Amplitude	Sand Grain Size
*K* values	1	0	2.98	1.95	1.1	1.01
	2	0.7	1.23	1.85	1.31	1.95
	3	1.86	1.67	1.24	1.13	1.14
	4	3.16	1.46	2.21	2.63	2.58
	5	3.32	1.7	1.79	2.87	2.36
*K*_avg_ values	1	0	0.6	0.39	0.22	0.2
	2	0.14	0.25	0.37	0.26	0.39
	3	0.37	0.33	0.25	0.23	0.23
	4	0.63	0.29	0.44	0.53	0.52
	5	0.66	0.34	0.36	0.57	0.47
Best level		15	5	8	140	0.7
Range *R*		0.52	0.35	0.19	0.35	0.31
Number of levels		4	5	5	5	5
Average number of repetitions for each level *r*		6	5	5	5	5
Convert coefficient *d*		0.45	0.4	0.4	0.4	0.4
Corrected range *R*′		0.58	0.31	0.17	0.32	0.28

**Table 8:** Multifactor analysis of variance results of the burial ratio.

	Quadratic Sum	*df*	Mean Square	*F*	*p*
Fish body length	1.723	4	0.431	50.942	0.001 **
Flapping frequency	0.372	4	0.093	10.998	0.02 *
Flapping number	0.101	4	0.025	2.996	0.157
Flapping amplitude	0.602	4	0.151	17.817	0.008 **
Sand grain size	0.401	4	0.1	11.852	0.017 *

Note: * indicates *p* < 0.05, ** indicates *p* < 0.01.

**Table 9:** Results of the range analysis of input power (Unit: MW).

Item	Level	Fish Body Length	Flapping Frequency	Flapping Number	Flapping Amplitude	Sand Grain Size
*K* values	1	1.86	54.87	20.75	36.54	58.53
	2	11.51	54.14	34.07	36.57	42.46
	3	29.90	30.32	54.25	55.14	39.79
	4	83.59	55.87	60.25	47.81	50.73
	5	122.97	54.63	80.51	73.77	58.32
*K*_avg_ values	1	0.37	10.97	4.15	7.31	11.71
	2	2.3	10.83	6.81	7.31	8.49
	3	5.98	6.06	10.85	11.03	7.96
	4	16.72	11.17	12.05	9.56	10.15
	5	24.59	10.93	16.10	14.75	11.66
Best level		3	7	2	60	0.5
Range *R*		24.22	5.11	11.95	7.45	3.75
Number of levels		5	5	5	5	5
Average number of repetitions for each level *r*		5	5	5	5	5

**Table 10:** Multifactor analysis of variance results for the input power.

	Quadratic Sum	*df*	Mean Square	*F*	*p*
Fish body length	2131.171	4	532.793	9.383	0.026 *
Flapping frequency	96.809	4	24.202	0.426	0.785
Flapping number	432.661	4	108.165	1.905	0.274
Flapping amplitude	191.552	4	47.888	0.843	0.564
Sand grain size	60.721	4	15.180	0.267	0.885

Note: * indicates *p* < 0.05.

**Table 11:** Results of the range analysis of burial efficiency.

Item	Level	Fish Body Length	Flapping Frequency	Flapping Number	Flapping Amplitude	Sand Grain Size
*K* values	1	0	2.6	4.22	1.65	1.11
	2	1.26	2.11	2.43	2.03	1.75
	3	3.40	2.55	0.69	1.1	1.84
	4	3.34	1.22	1.83	2.67	2.92
	5	2.4	1.92	1.23	2.95	2.78
*K*_avg_ values	1	0	0.52	0.84	0.33	0.22
	2	0.25	0.42	0.49	0.41	0.35
	3	0.68	0.51	0.14	0.22	0.37
	4	0.67	0.24	0.37	0.53	0.58
	5	0.48	0.38	0.25	0.59	0.56
Best level		9	5	2	140	0.7
Range *R*		0.43	0.28	0.71	0.37	0.36
Number of levels		4	5	5	5	5
Average number of repetitions for each level *r*		6	5	5	5	5
Convert coefficient *d*		0.45	0.4	0.4	0.4	0.4
Corrected range *R*′		0.47	0.25	0.63	0.33	0.32

**Table 12:** Multifactor analysis of variance results for the burial efficiency.

	Quadratic Sum	*df*	Mean Square	*F*	*p*
Fish body length	1.686	4	0.422	1.781	0.295
Flapping frequency	0.251	4	0.063	0.266	0.886
Flapping number	1.484	4	0.371	1.567	0.337
Flapping amplitude	0.451	4	0.113	0.476	0.755
Sand grain size	0.461	4	0.115	0.487	0.749

## Data Availability

The data that support the findings of this study are available from the corresponding author upon reasonable request. No publicly archived datasets were generated or analyzed in this study.
